# Repulsive guidance molecule acts in axon branching in *Caenorhabditis elegans*

**DOI:** 10.1038/s41598-021-01853-8

**Published:** 2021-11-16

**Authors:** Kaname Tsutsui, Hon-Song Kim, Chizu Yoshikata, Kenji Kimura, Yukihiko Kubota, Yukimasa Shibata, Chenxi Tian, Jun Liu, Kiyoji Nishiwaki

**Affiliations:** 1 Department of Bioscience, Kwansei Gakuin University, 2-1 Gakuen, Sanda, 669-1337 Japan; 2 Department of Molecular Biology and Genetics, Cornell University, Ithaca, NY 14853 USA

**Keywords:** Developmental biology, Neuroscience

## Abstract

Repulsive guidance molecules (RGMs) are evolutionarily conserved proteins implicated in repulsive axon guidance. Here we report the function of the *Caenorhabditis elegans* ortholog DRAG-1 in axon branching. The axons of hermaphrodite-specific neurons (HSNs) extend dorsal branches at the region abutting the vulval muscles. The *drag-1* mutants exhibited defects in HSN axon branching in addition to a small body size phenotype. DRAG-1 expression in the hypodermal cells was required for the branching of the axons. Although DRAG-1 is normally expressed in the ventral hypodermis excepting the vulval region, its ectopic expression in vulval precursor cells was sufficient to induce the branching. The C-terminal glycosylphosphatidylinositol anchor of DRAG-1 was important for its function, suggesting that DRAG-1 should be anchored to the cell surface. Genetic analyses suggested that the membrane receptor UNC-40 acts in the same pathway with DRAG-1 in HSN branching. We propose that DRAG-1 expressed in the ventral hypodermis signals via the UNC-40 receptor expressed in HSNs to elicit branching activity of HSN axons.

## Introduction

Axon branching is a fundamental process for proper axon projection to target tissues and for the formation of correct synapses, both of which are important for development of the functional neural network. Axon branching begins with the formation of an actin-rich filopodium from the existing axon followed by extension of microtubules along the actin filaments^1^. Formation of filopodia and subsequent neurite extension involve various regulators for actin and microtubule polymerization and bundling. The location and the polarity of axon branching are dictated by extracellular cues, along with cytoskeletal activities. Axon guidance molecules are involved in this process.

Netrin-1 is a secreted guidance molecule that induces local filopodial protrusions in the axon shaft, which give rise to branches in the cortical neurons. In contrast, SEMA3A represses cortical axon branching^2^. Ephrins are membrane-bound molecules that abolish branching of thalamic axons^3^. In addition to these well-known guidance molecules, repulsive guidance molecules (RGMs) also repress axon branching in cortical neurons and mossy fibers of the hippocampus^4,5^. In vertebrates, RGMs are glycosylphosphatidylinositol (GPI)-linked membrane proteins that constitute a family with four members: RGMa, RGMb (DRAGON), RGMc (hemojuvelin), and RGMd^6^. RGMa was first discovered as an axon guidance cue that has a repulsive activity to retinal axons. RGMa is expressed in the embryonic tectum in an anterior-to-posterior concentration gradient and functions during the development of the retinotectal projection^7^.

By binding to the membrane receptor neogenin, RGMs function in axon guidance and neuronal survival as well as inhibition of axonal regeneration^8–10^. RGMs also bind bone morphogenetic proteins (BMPs) in the regulation of iron homeostasis and endochondral bone development^11–13^. Although the function of RGMs in axon guidance as a result of growth cone repulsion has been well studied, their role in axon branching is still elusive. Because of the lethality of knock-out mice and the functional redundancy of RGM proteins, the functions of RGMs have been mostly analyzed using in vitro culture systems^14^.

The nematode *C. elegans* has a single ortholog of RGM, DRAG-1. Loss-of-function mutations in *drag-1* result in a small body size as well as genetic suppression of the mesodermal coelomocyte loss phenotype of *sma-9* mutants^15^. *drag-1* function in the hypodermal cells is required for the control of body size, while *drag-1* function in the mesodermal M cell lineage controls coelomocyte differentiation^15,16^. In the present study, we showed that *drag-1* expressed in the hypodermis functions in the formation of branches of hermaphrodite-specific neurons (HSNs), which innervate egg-laying muscles of the vulva^17^. Small body size mutants *sma-1* and *sma-5* also exhibited HSN axon branching defects although they act in genetic pathways distinct from that of *drag-1*. Genetic analyses suggested that *unc-40* acts in the same pathway as *drag-1*. Because DRAG-1 binds the receptor UNC-40^16^ and UNC-40 is expressed in HSNs^18^, our findings suggest that DRAG-1 acts on the receptor UNC-40 to induce axon branching of HSNs.

## Results

### *drag-1* mutants are defective in axon branching of HSNs

**Figure 1:**
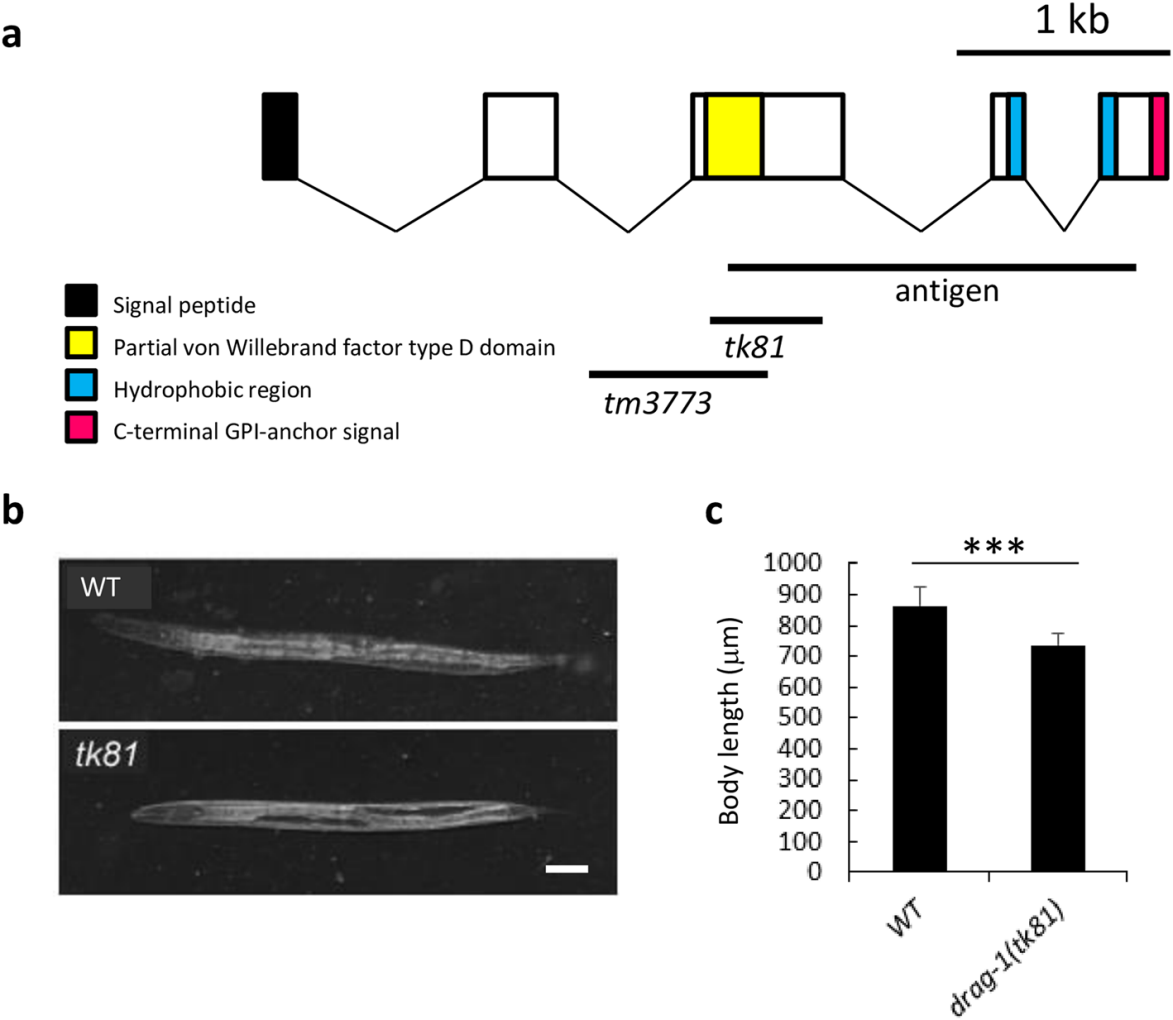
Gene structure and mutant phenotype of *drag-1* animals. (**a**) Structure of *drag-1* and mutation sites of the *tk81* and *tm3773* alleles. The exon and intron regions were determined by sequencing cDNA generated from isolated *drag-1* mRNA. Black, yellow, blue, and magenta boxes indicate N-terminal signal peptide, partial von Willebrand factor type D domain, hydrophobic region, and C-terminal GPI-anchor signal sequence, respectively^15^. Bars depict the region of the cDNA used for expressing the antigenic peptide for producing antibodies and the respective mutation sites. *tk81* is a 494-bp deletion within exon 3, which is expected to produce a truncated polypeptide that is missing the C-terminal 278 amino acids. *tm3773* is an 892-bp deletion spanning from intron 2 to exon 3 (WormBase). (**b**) Body length phenotype of *drag-1(tk81)* mutants. Body length of young adult hermaphrodites. *tk81* mutants had shorter bodies compared with wild type. Anterior is to the left. Scale bar: 50 μm. (**c**) Quantification of body length of young adult hermaphrodites for wild-type and *drag-1* mutant animals. Significant difference was determined by Student’s t-test. ***P < 0.001. n = 60 for each strain.

**Figure 2:**
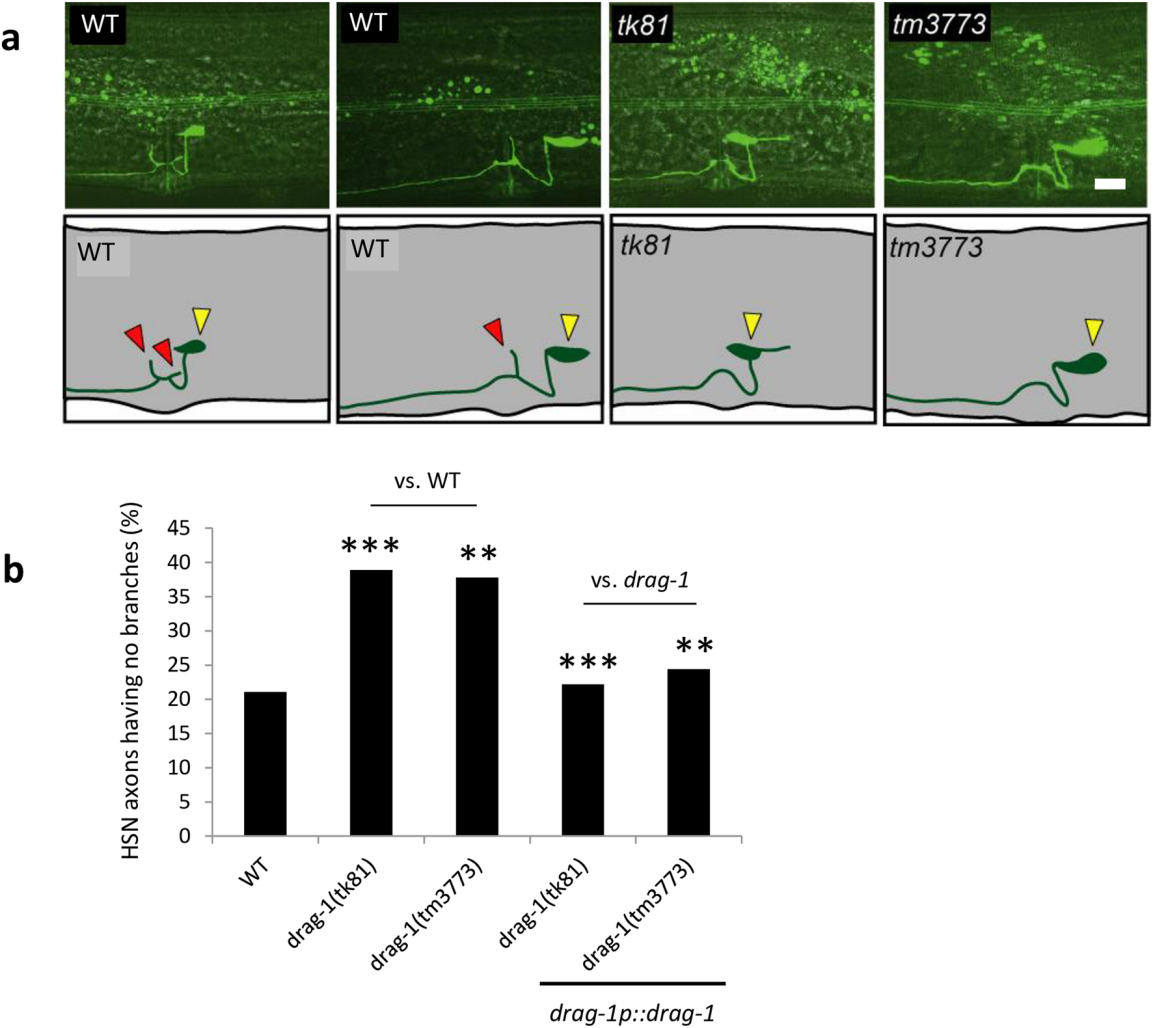
HSN branching phenotypes. (**a**) HSN axon branching. Upper panels: Confocal micrographs of HSNs in wild-type and *drag-1* mutant young-adult hermaphrodites with the *unc-86p::myrGFP* transgene. Lower panels: Schematic representations of HSN morphology. Yellow and red arrowheads depict the HSN cell body and axonal branches, respectively. Anterior is to the left, dorsal top. Scale bar: 10 μm. (**b**) Quantification of the HSN branching phenotypes in various strains. Percentages of HSNs having no branches are shown. Branching phenotypes were scored using fluorescence microscopy. *drag-1(tk81)* and *drag-1(tm3773)* mutants were compared with those transgenic for *drag-1p::drag-1*. Significant differences were determined by Fisher’s exact test. ***P < 0.001, **P < 0.01. n = 180 for each strain.

*drag-1* encodes the sole *C. elegans* ortholog of the RGM family of proteins. We isolated a deletion allele of *drag-1*, *tk81* (Fig. 1a). *drag-1(tk81)* animals had a smaller body size compared to wild type. The *drag-1(tm3773)* deletion mutant obtained from the National Bioresource Project showed a similar phenotype (Fig. 1a–c, Supplementary Fig. S1a,b). Because RGM family proteins act in axon guidance in mammals, we examined axonal morphology using a pan-neuronal GFP reporter *ncIs2*^19^. We found no gross defects in axon trajectories in the mutants compared with wild type. We then examined the morphology of the axon of hermaphrodite specific neurons (HSNs) using *kyIs262[unc-86p::myrGFP; odr-1::RFP]*^20^ as a transgenic marker. In wild type animals, the cell bodies of the bilateral HSNs are positioned slightly posterior to the vulva. They extend a single axon toward the ventral nerve cord. After reaching the nerve cord, the axon is redirected dorsally and anteriorly and reaches the lateral position of the developing vulva, where it turns again, this time in a ventral and anterior direction toward the ventral nerve cord^21^. The projection of HSN axons was not affected in the *drag-1* mutants (Supplementary Table S1). Although the axon usually sprouts one or two dorsal branches at the vulva in the wild type, the number of axons with branches was significantly reduced in the *drag-1* mutants (Fig. 2a,b). To examine whether the branching defect is caused by loss of *drag-1* function, we introduced a plasmid containing a fragment of the wild-type gene (*drag-1p::drag-1*) into the *drag-1* mutants as a transgene. The transgene fully rescued the branching defect (Fig. 2b), confirming the function of DRAG-1 in HSN axon branching. Because *drag-1* mutants did not accumulate fertilized eggs in the uterus, they were not egg-laying defective.

### *drag-1* acts in parallel pathways with *sma-1* and *sma-5*

**Figure 3:**
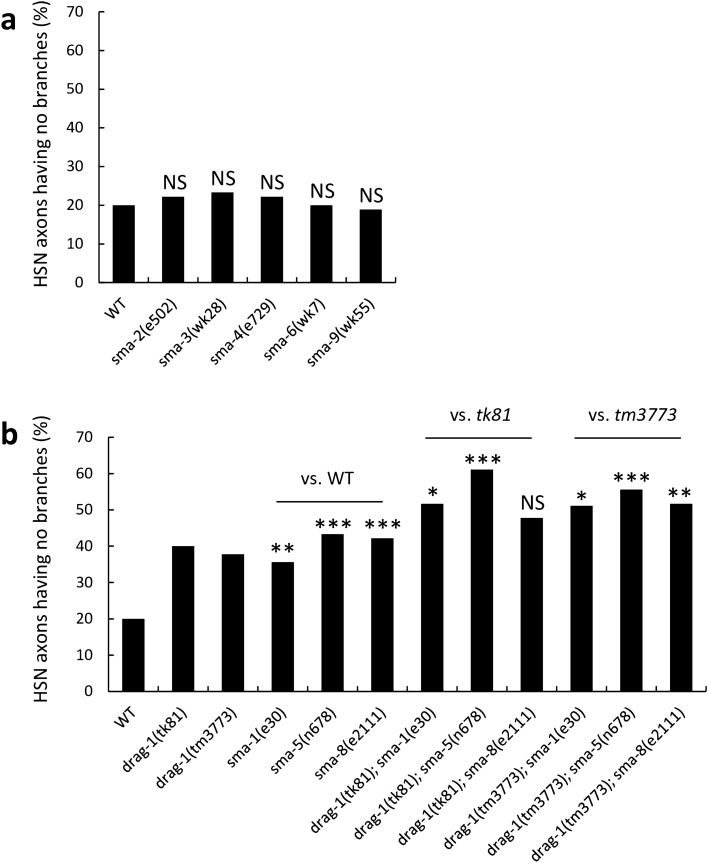
Genetic interactions between *drag-1* mutants and *sma* mutants. Percentages of HSNs having no branches are shown. (**a**) *sma-2(e502)*, *sma-3(wk28)*, *sma-4(e729)*, *sma-6(wk7)*, and *sma-9(wk55)* mutants were compared with wild type. (**b**) *drag-1(tk81)* and *drag-1(tm3773)* mutants were compared with *sma-1(e30)*, *sma-5(n678)*, and *sma-8(e2111)* mutants and with double mutants consisting of *drag-1(tk81)* or *drag-1(tm3773)* in combination with individual *sma* mutations. Significant differences were determined by Fisher’s exact test. ***P < 0.001, **P < 0.01, *P < 0.05. *NS* not significant. n = 180 for each strain.

Because *drag-1* mutants result in a small body size (Sma) phenotype, we examined whether other *sma* mutants affect HSN axon branching. Among the eight *sma* mutants examined, *sma-2(e502)*, *sma-3(wk28)*, *sma-4(e729)*, *sma-6(wk7)*, and *sma-9(wk55)* did not show HSN branching defects (Fig. 3a). We found HSN branching defects similar to that observed in the *drag-1* mutants in *sma-1(e30)*, *sma-5(n678)*, and *sma-8(e2111)* (Fig. 3b). *sma-1* and *sma-5* encode β_H_-spectrin and MAP kinase, respectively^22,23^. *sma-8(e2111)* is a dominant mutation for which the causative gene has not yet been identified. We produced double mutants between *drag-1* mutants and these *sma* mutants and found that all double mutants exhibited HSN branching defects stronger than those observed in the respective single mutants (Fig. 3b). Because *drag-1(tk81)* and *drag-1(tm3773)* mutants are putative null alleles, these results suggested that *sma-1* and *sma-5* act in pathways different from that of *drag-1* to regulate HSN branching. Because of the dominancy of the *sma-8(e2111)* mutation, the relationship between *drag-1* and *sma-8* remains to be determined.

### GPI anchoring of DRAG-1 is important for HSN branching

**Figure 4:**
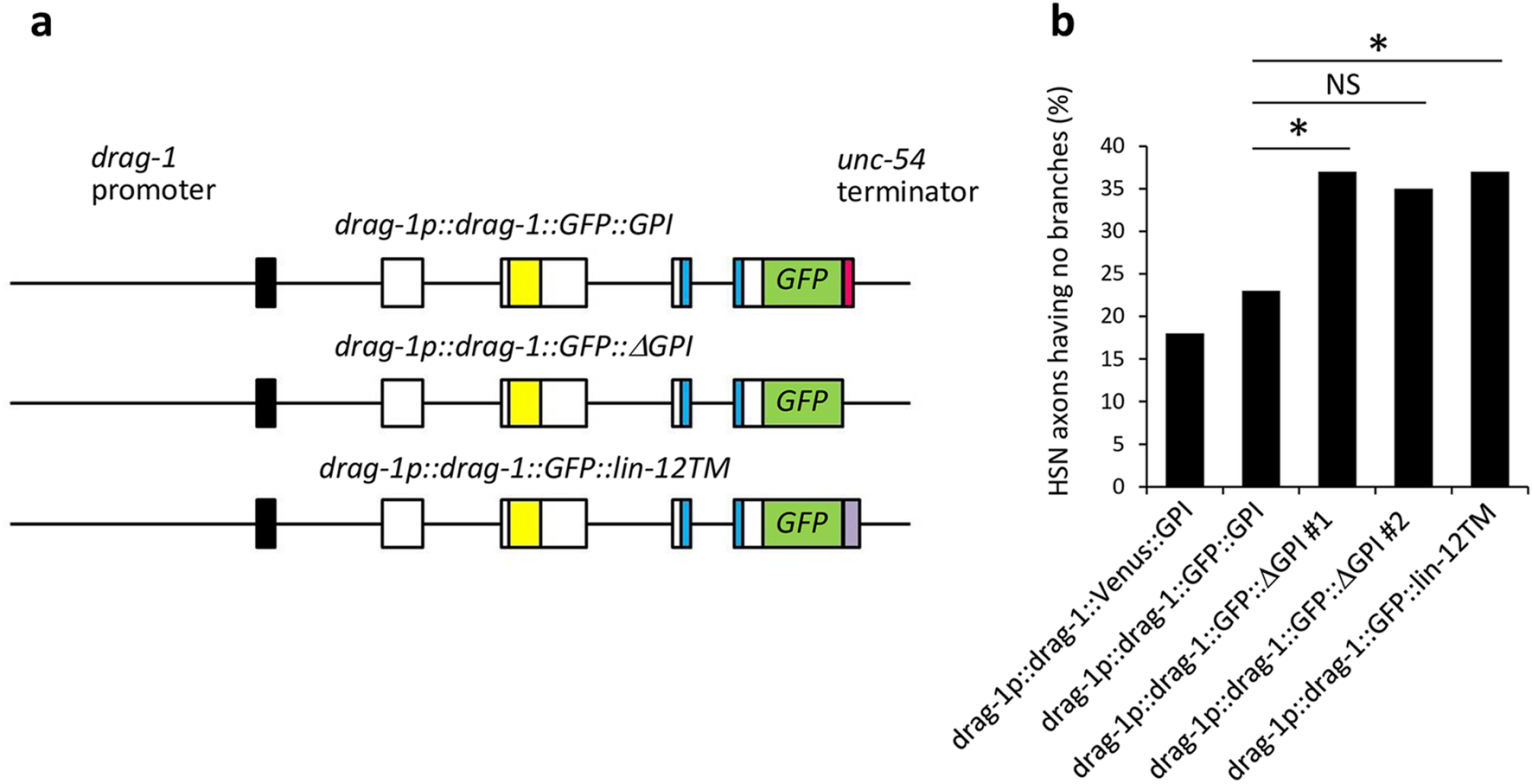
Rescue experiments of *drag-1* mutants with modified DRAG-1 proteins. (**a**) Schematic representation of the GFP fusion constructs. The GFP or Venus coding sequence was inserted between amino acid (aa) 395 and 396 of the *drag-1* coding region, just prior to the cleavage site of the C-terminal pro-peptide for *drag-1p::drag-1::GFP::GPI*. The C-terminal GPI-anchor signal (aa 387–408) was deleted from *drag-1p::drag-1::GFP::GPI* for *drag-1p::drag-1::GFP::*∆*GPI*. The *lin-12* transmembrane domain (aa 907–934) (shown in purple) was connected with *drag-1p::drag-1::GFP::*∆*GPI* for *drag-1p::drag-1::GFP::lin-12TM*^15^*.* (**b**) Percentages of HSNs having no branches are shown for the *drag-1(tk81)* mutants expressing the different rescuing constructs shown in (**a**). Data for *drag-1p::drag-1::Venus::GPI* is also shown. Significant differences were determined by Fisher’s exact test*.* *P < 0.05. *NS* not significant. n = 180, 107, 105, 91, and 109 for *drag-1p::drag-1::Venus::GPI*, *drag-1p::drag-1::GFP::GPI*, *drag-1p::drag-1::GFP::∆GPI #1*, *drag-1p::drag-1::GFP::∆GPI #2*, and *drag-1p::drag-1::GFP::lin-12TM*, respectively. The *#1* and *#2* refer to two independently isolated transgenic lines.

Because DRAG-1 is thought to be modified by a GPI-anchor, we placed the GFP or Venus coding region right upstream of the putative cleavage site of the C-terminal pro-peptide sequence (*drag-1p::drag-1::GFP::GPI* or *drag-1p::drag-1::Venus::GPI*) to keep the GPI-anchor signal intact. These constructs rescued the branching defect of the mutants (Fig. 4a,b). We examined whether the GPI anchoring is important for DRAG-1 to act in axon branching. A construct with the deleted C-terminal GPI-anchor signal sequence (*drag-1p::drag-1::GFP::∆GPI*) failed to rescue the branching defects of *drag-1* mutants (Fig. 4a,b). We also examined a construct in which the C-terminal GPI-anchor signal was replaced by the transmembrane domain of the LIN-12/Notch receptor (*drag-1p::drag-1::GFP::lin-12TM*). This chimeric protein, which is potentially localized to the plasma membrane, failed to rescue the mutant defects (Fig. 4a,b). These results suggested that DRAG-1 needs to be anchored to the plasma membrane by the GPI anchor rather than being embedded as a transmembrane protein.

### Expression of DRAG-1

**Figure 5:**
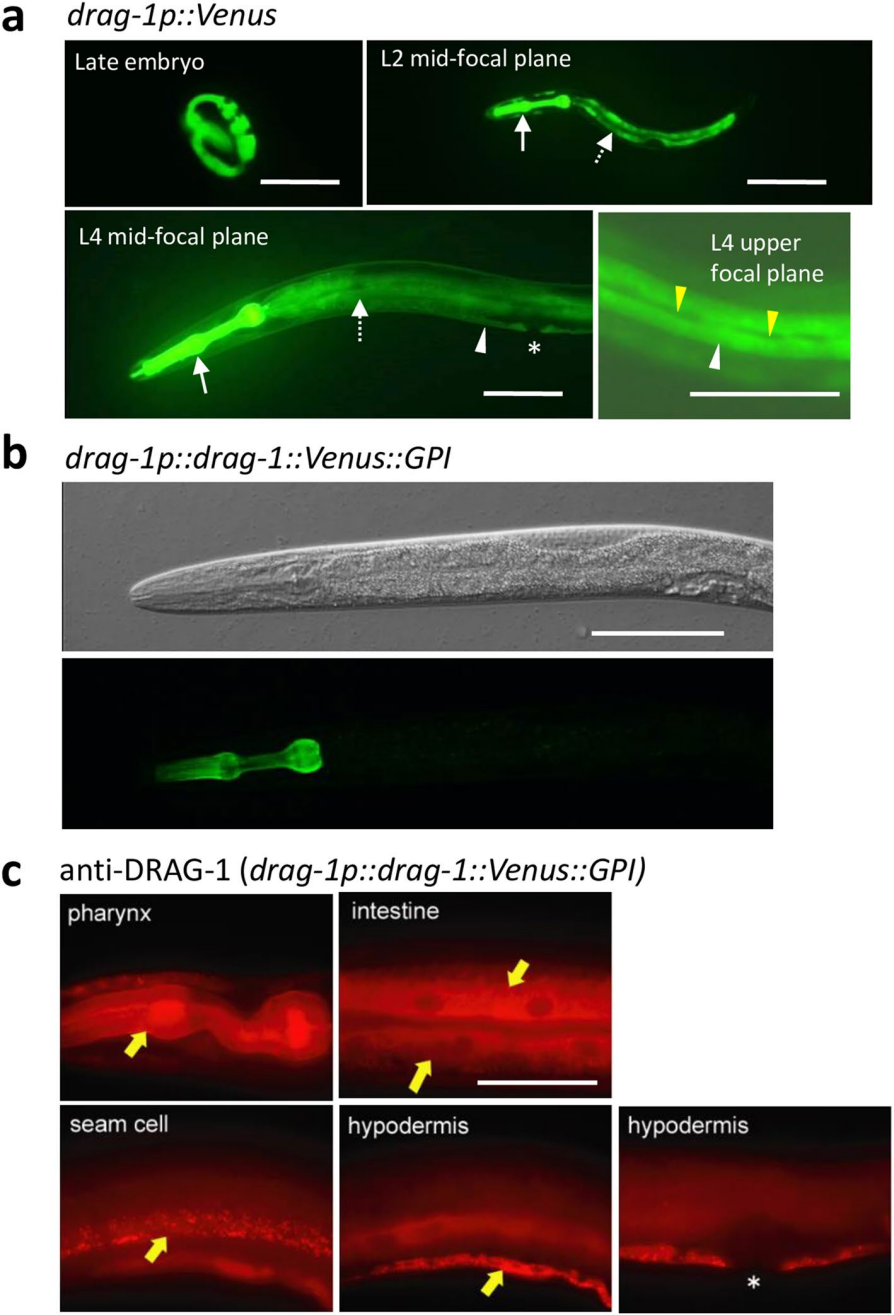
*drag-1* expression. (**a**) *drag-1p::Venus* expression. Expression was detected from late embryos to the adult stage in the pharynx, intestine, and hypodermis. White arrows, dotted arrows and white arrowheads correspond to the pharynx, intestine and hypodermis, respectively. Yellow arrowheads and asterisk point to seam cells and developing vulval epithelium which do not express Venus, respectively. Scale bar: 100 μm. (**b**) Expression of *drag-1p::drag-1::Venus::GPI.* The *drag-1p::drag-1::Venus::GPI* plasmid was injected into *unc-119(e2498)* animals at 150 ng/μl with 30 ng/μl of pBSII KS(–) and 20 ng/μl pDP#MM016B. DIC (upper) and fluorescence (lower) images of an L4 stage animal are shown. Venus expression was detected only in the pharynx. Anterior is to the left. Scale bar: 100 μm. (**c**) Immunostaining using anti-DRAG-1. L4 to young-adult animals expressing *drag-1p::drag-1::Venus::GPI* were fixed and stained with anti-DRAG-1. DRAG-1 expression was detected in the pharynx, intestine, hypodermal seam cells, and ventral hypodermal cells (arrows) except the vulval hypodermis (asterisk). Scale bar: 50 μm.

To understand how DRAG-1 functions, we examined its expression pattern using both transcriptional and translational reporters, as well as immunostaining using anti-DRAG-1 antibodies that we generated. Expression of a transcriptional *drag-1p::Venus* reporter was detected in the pharynx, intestine and the syncytial hypodermis from late embryos to adult stages. Notably, Venus expression was not detected in hypodermal seam cells and the vulval hypodermis (Fig. 5a). Unlike the transcriptional reporter described above, we could only detect Venus expression in the pharynx using the functional translational fusion construct *drag-1p::drag-1::Venus::GPI* (Fig. 5b). We therefore raised polyclonal antibodies against a DRAG-1 peptide corresponding to amino acids 130–368 (Fig. 1a). Immunostaining experiments were performed using the L4 to young adult stage animals in which HSN branches are formed^24^. The antibodies detected no signals in non-transgenic wild-type animals, but detected signals in the pharynx, intestinal cells, hypodermal seam cells, and ventral hypodermal cells (except in the vulval region) in animals transgenic for either *drag-1p::drag-1* or *drag-1p::drag-1::Venus::GPI*. (Fig. 5c). The expression patterns were consistent with the observation using anti-GFP in animals expressing *drag-1p::drag-1::GFP::GPI*^15^. Thus, it is likely that the level of expression of endogenous DRAG-1 is low. Alternatively, DRAG-1 expression might be upregulated only transiently in HSN branch formation. The hypodermal signals detected by the anti-DRAG-1 antibodies appeared in a granular pattern in the cytoplasm. This could be due to the accumulation of DRAG-1 in the ER or Golgi apparatus as a result of over-expression of *drag-1*. We detected no DRAG-1 expression in the nervous system including HSNs even in the over-expressed condition.

### DRAG-1 functions in hypodermal cells for axon branching

**Figure 6:**
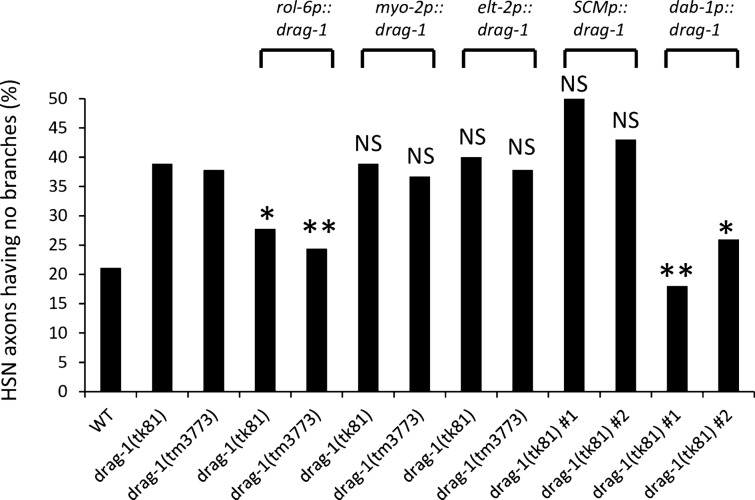
Tissue-specific rescue experiments of *drag-1* mutants. Percentages of HSNs having no branches are shown. *drag-1(tk81)* and *drag-1(tm3773)* mutants were compared with those transgenic for *rol-6p::drag-1*, *myo-2p::drag-1*, *elt-2p::drag-1*, *SCMp::drag-1 and dab-1p::drag-1*. Two independently isolated transgenic lines (*#1* and *#2)* were examined for each transgene. Significant differences were determined by Fisher’s exact test relative to the corresponding *drag-1* mutants. **P < 0.01, *P < 0.05. *NS* not significant. n = 180 for each strain.

We have shown that *drag-1* is important for axon branching, but *drag-1* is not expressed in the nervous system. To determine the tissues in which DRAG-1 expression is important for axon branching, we expressed *drag-1* under tissue-specific promoters. We found that hypodermal expression of DRAG-1 using the *rol-6* promoter (*rol-6p::drag-1*)^25^ rescued the branching defect, whereas expression in the pharyngeal muscle (*myo-2p::drag-1*)^26^ or in the intestine (*elt-2p::drag-1*)^27^ did not (Fig. 6). These results indicated that DRAG-1 functions in hypodermal cells to induce axon branching of the HSNs. Because we detected DRAG-1 expression in both ventral hypodermis and lateral hypodermal seam cells, we tested whether the expression in the seam cells could rescue the HSN branching defect of *drag-1* mutants. *drag-1* expression under the seam cell specific SCM promoter^28^ failed to rescue the HSN branching defect (Fig. 6). We also attempted to specifically express *drag-1* in the ventral hypodermis, but we failed to find an appropriate promoter for this purpose. To circumvent this problem, we drove the expression of *drag-1* under the *dab-1* promoter, which drives gene expression in the developing vulval precursor cells^29^. Interestingly, the *dab-1p::drag-1* construct successfully rescued the branching defect (Fig. 6). Thus even though *drag-1* is not normally expressed in the vulval precursor cells, forced expression of *drag-1* in these cells is sufficient for HSN branching, again supporting a role of DRAG-1 functioning in hypodermal cells for axon branching.

### *drag-1* acts in the same pathway as *unc-40* in HSN axon branching

**Figure 7:**
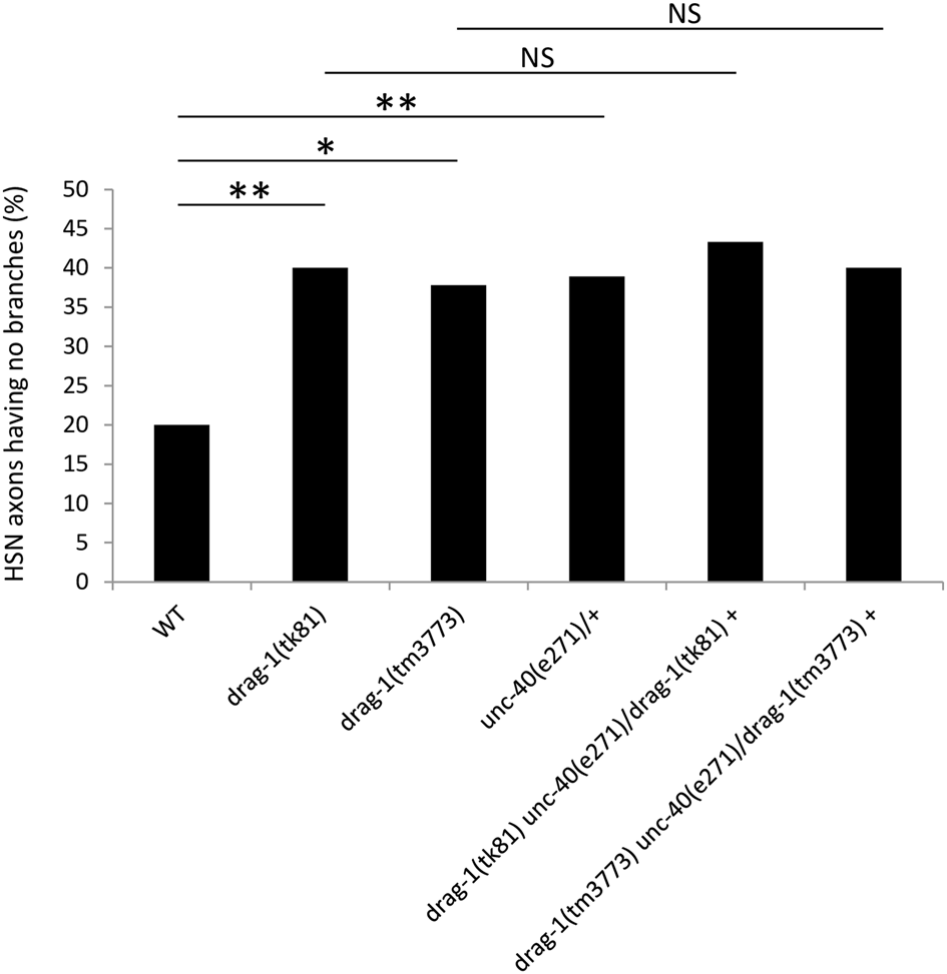
*drag-1* does not enhance *unc-40/+*with respect to HSN branching defects. Percentages of HSNs having no branches are shown. *drag-1(tk81)* and *drag-1(tm3773)* mutants were compared with *unc-40(e271)/+*heterozygotes and with *drag-1(tk81) unc-40(e271)/+*and *drag-1(tm3773) unc-40(e271)/+*double mutants. Significant differences were determined by Fisher’s exact test. **P < 0.01, *P < 0.05. *NS* not significant. n = 90 for each strain.

Neogenin is a receptor for RGMa for axonal growth cone guidance^8,30^ and UNC-40 is the sole ortholog of neogenin in *C. elegans*. We examined the HSN axons of *unc-40(e271)* null allele. The axons of the *unc-40* mutants mostly failed to extend to the ventral side and the branches were rarely observed at the vulva or other locations (Supplementary Fig. S2, Table S1). However, we observed branching defects in *unc-40(e271)/+*heterozygotes with similar penetrance as in the *drag-1* mutants. *unc-40(e271)/+*heterozygotes exhibited no abnormality in HSN axon trajectory (Supplementary Table S1). The branching defect in unc-*40(e271)/+*was not enhanced when combined with *drag-1* null mutants (Fig. 7). Therefore, UNC-40 acts in the same genetic pathway as DRAG-1 in HSN axon branching.

## Discussion

In this study, we have shown that the sole *C. elegans* repulsive guidance molecule DRAG-1 functions in hypodermal cells to regulate axon branching of the HSNs that form synapses with the vulval egg-laying muscles in hermaphrodites. We further showed that DRAG-1 functions in the same genetic pathway as the neogenin homolog UNC-40 in regulating axon branching.

DRAG-1 has been previously shown to function in the BMP signaling pathway to regulate body size and mesoderm development^15^. *sma-6* (BMP type I receptor); *sma-2*, *-3*, and *-4* (Smads); and *sma-9* (BMP antagonist schnurri) are components of the BMP signaling pathway in *C. elegans*. Mutations in these genes result in a small body size (Sma) phenotype. However, none of the *sma* mutants in the BMP pathway affected HSN axon branching. Instead, we observed HSN axon branching defects in *sma-1(e30)* and *sma-5(n678)* mutants, which are not involved in the BMP signaling. These results suggest that BMP signaling does not regulate HSN axon branching and that the function of DRAG-1 in regulating HSN axon branching is independent of BMP signaling.

Unlike BMP signaling, two genes known to function in regulating body size, *sma-1* and *sma-5*, also play a role in regulating HSN axon branching. We have shown that SMA-1 and SMA-5 appear to act in pathways parallel to that of DRAG-1. *sma-1* encodes β_H_-spectrin, which is a very large spectrin found in invertebrates such as *C. elegans* and *Drosophila*^22^. The submembrane skeletal network is primarily formed from α2β2 spectrin tetramers, each composed of two α-spectrin and two β-spectrin subunits^31^. The spectrin network interacts with peripheral actin filaments to act in synapse function, muscle sarcomere structure, and axonal outgrowth^32,33^. Although SMA-1 function in shaping cells in the hypodermis and pharyngeal muscles has been reported^34,35^, its function in neuronal cells is unknown. SMA-1 may function in HSNs for branch formation by regulating the actin filaments. SMA-5/MAP kinase is specifically expressed in the intestine to control intestinal tube stability and body size^23^. Because the intestine has no direct contact with HSNs, it is possible that SMA-5 indirectly affects branching of the neuron. Because 60% of HSNs produce at least one branch in the *drag-1* null mutant background and 40–50% of HSNs still make branches even in *drag-1; sma-1* or *drag-1; sma-5* double mutants, additional mechanisms must exist to control branch formation in HSNs. These additional factors/pathways include KAL-1/anosmin-1 and EGL-17/fibroblast growth factor (FGF), which act through SAX-7/L1CAM and EGL-15/FGF receptor to regulate HSN branching^36^. Additionally, the immunoglobulin superfamily protein SYG-1, which is expressed in HSN, is required for HSN branching and appropriate synaptogenesis with egg-laying muscles^37^.

How does DRAG-1 functions to regulate HSN axon branching? We believe that hypodermally expressed DRAG-1 acts through the neogenin receptor UNC-40 that is expressed in the HSNs to regulate HSN axon branching. We found via immunostaining that DRAG-1 is expressed in hypodermal seam cells and in the ventral hypodermis with the exception of the vulval epithelium. Furthermore, the defective axon branching of *drag-1* mutants can be rescued by expressing *drag-1* under the hypodermis specific *rol-6* promoter or under the vulval epithelial precursor cell-specific *dab-1* promoter, but not the hypodermal seam cell-specific SCM promoter. The *dab-1* promoter drives gene expression in the descendants of vulval precursor cells P5.p, P6.p and P7.p during the Pn.pxx stage and in the descendants of P5.p and P7.p during the Pn.pxxx stage^29^. It is during this period that the HSN axons contact with some of these vulval precursor cells, defasciculate from the ventral nerve cord and form branches^24,37,38^. Thus, it is likely that DRAG-1 ectopically expressed in the vulval precursor cells signals the HSN axon to induce branching. In wild type animals DRAG-1 is expressed in the ventral hypodermis, which is directly adjacent to the ventral nerve cord, and the HSN axons fasciculate with the ventral nerve cord twice at regions posterior and anterior to the vulva^21^. Thus, DRAG-1 from the hypodermal cells can bind to the neogenin receptor UNC-40 that is expressed in the HSNs to induce HSN branching in a non-cell-autonomous fashion. This is consistent with UNC-40 being an ortholog of vertebrate neogenin, which acts as a receptor for RGM proteins^30,39^. Moreover, DRAG-1 physically interacts with the extracellular domain of UNC-40 in *C. elegans*^16^, similar to the interaction observed between human RGMc and neogenin^40^. Neither DRAG-1 with its GPI-anchor sequence deleted (therefore as a potential secreted form) nor DRAG-1 fused with the LIN-12 transmembrane domain (therefore as a potential membrane-anchored form) rescued the axon branching defects of *drag-1* mutants. The latter was unexpected because the same construct significantly rescues the *drag-1* defect in the control of mesodermal cell differentiation^15^. With respect to mesodermal cell differentiation, DRAG-1 and UNC-40 are expressed and function in the same cells to promote BMP signaling. In HSN branching, however, they are expressed in different cells that contact each other. Thus, it is possible that the modes of signaling from DRAG-1 to UNC-40 may differ depending on the cellular context. Studies of vertebrate RGM and neogenin proteins have yielded similar findings showing that they either function in the same cells or in different cells to regulate different processes^14^.

In summary, we provide in vivo evidence that RGM proteins function to promote axon branching in *C. elegans*. Our finding contrasts with the observation that RGM proteins suppress the branching of axons in the mammalian brain^4,5^. RGMs may function in both ways depending on the tissues or the phases of organogenesis. Further research is needed to understand the precise function of RGMs in axon branching.

## Methods

### Strains and culture conditions

Culture and handling of *C. elegans* were as described^41^. The following strains were used: N2 (wild type, WT), *drag-1(tk81)* (this work)*, drag-1(tm3773)* (National Bioresource Project)*, unc-119(e2498)*^42^, *sma-1(e30)*, *sma-2(e502)*, *sma-3(wk28)*, *sma-4(e729)*, *sma-5(n678)*, *sma-6(wk7), sma-8(e2111), sma-9(wk55), unc-40(e271)*^41,43–45^. HSNs were visualized using an integrated transgene *kyIs262[unc-86p::myrGFP; odr-1::RFP]*^20^. *drag-1(tk81)* was isolated by the trimethylpsoralen and UV irradiation method^46^.

### Analysis of HSN branching

HSN branching phenotype was analyzed with confocal or fluorescence microscopy using young adult hermaphrodites. Young adult hermaphrodites were selected from the mixed population by having mature vulva and no fertilized eggs in the uterus.

### Plasmid construction

*drag-1p::drag-1::GFP::GPI*, *drag-1p::drag-1::GFP::∆GPI*, and *drag-1p::drag-1::GFP::lin-12TM* correspond to pJKL849, pCX192, and pCX194, respectively^15^. To produce *drag-1p::Venus*, the *drag-1* promoter region was PCR amplified from genomic DNA using primers 5′-TAGCCTGCAGGTTTCCGAAGACAGGGGAACATGGAA-3′ and 5′-GTTCGTCGACACTCTGTCAAGTCTTCTCATCTCACG-3′, digested with *Pst*I and *Sal*I, and cloned into the *Pst*I and *Sal*I sites of pPD95.75. To produce *drag-1p::drag-1::Venus*, the *drag-1* coding region was PCR amplified from genomic DNA with primers 5′-GTCAGTCGACATGTCAATAGTCTATCTCG-3′ and 5′-CATGGGTACCAAGCATAACAATGATAAAAGAGC-3′, digested with *Sal*I and *Kpn*I, and cloned into the *Sal*I and *Kpn*I sites of *drag-1p::Venus*. To produce *drag-1p::drag-1*, *drag-1p::drag-1::Venus* was PCR amplified with primers 5′-GATCGCTAGCCTTGTCTGGTGTCAAAAATAATAGG-3′ and 5′-TCGCTAGCTCAGCATAACAATGATAAAAGAGCAAAA-3′, digested with *Nhe*I, and self-ligated. To produce *drag-1p::drag-1::Venus::GPI*, the Venus coding region was PCR amplified from pPD95.75 with primers 5′-GCATGGGCCCAGGGTACCGGTAGAAAAAATGAGT-3′ and 5′-GCATGGGCCCTTTGTATAGTTCATCCATGCCAAG-3′, digested with *Apa*I, and ligated into *drag-1p::drag-1::GFP::GPI* (pJKL849) in which the green fluorescent protein (GFP) coding region had been deleted by *Apa*I digestion. *rol-6* and *myo-2* promoter regions were PCR amplified with primers 5′-CAGTGCATGCCGAGAAGAGTCCGGTGTGAA-3′ and 5′-CAGTGTCGACCTGGAAATTTTCAGTTAGATCTAAAG-3′, and 5′-CAGTGCATGCGTGAGCAAGTGTGCGGCATC-3′ and 5′-CAGTGTCGACTTCTGTGTCTGACGATCGAGGG-3′, respectively. These PCR fragments were digested with *Sph*I and *Sal*I and ligated with *drag-1p::drag-1* in which the *drag-1* promoter region was deleted by *Sph*I and *Sal*I digestion to produce *rol-6p::drag-1*, *myo-2p::drag-1*. *elt-2* promoter region was PCR amplified from genomic DNA with primers 5′-CAGTCCTGCAGGGTGACCGCTCAAAATAAAAGG-3′ and 5′-CAGTCTCGAGTCTATAATCTATTTTCTAGTTTCTA-3′. The PCR fragment was digested with *Sbf*I and *Xho*I and ligated with *drag-1p::drag-1* in which the *drag-1* promoter region was deleted by *Sbf*I and *Sal*I digestion to produce *elt-2p::drag-1*. To produce *SCMp::drag-1,* the SCM region was PCR amplified from genomic DNA with primers 5′-ATGAAATAAGCTTGCATGCCTGCAGCCAAGCTTGCATGCCTGCAG-3′ and 5′-CGAGATAGACTATTGACATGTCGACTCCTTTGGCCAATCCCGGG-3′, and the resulted fragment was fused with *drag-1p::drag-1* in which the *drag-1* promoter region was deleted by *Pst*I and *Sal*I digestion using In-Fusion Cloning Kit (Takara Bio). To produce *dab-1p::drag-1,* the *dab-1* promoter region was PCR amplified from genomic DNA with primers 5′-AAATAAGCTTGCATGCAAGATTATCCCAAATTGTGGACCGT-3′ and 5′-CTATTGACATGTCGACTGTTTCGAGAGAACCTTTAGAAATAAGATT-3′, and the resulted fragment was fused with *drag-1p::drag-1* in which the *drag-1* promoter region was deleted by *Sph*I and *Sal*I digestion using In-Fusion Cloning Kit (Takara Bio).

### Production of transgenic animals

We injected DNA mixtures into the gonads of *unc-119(e2498)*, *drag-1(tk81); unc-119(e2498); kyIs262 or drag-1(tm3773); unc-119(e2498); kyIs262* adult hermaphrodites^47^. For transgenic rescue experiments, test plasmids were injected at 10–20 ng/μl with 160–170 ng/μl of pBSII KS(–) and 20 ng/μl of *unc-119*^+^ plasmid pDP#MM016B^42^. For immunohistochemistry, *drag-1p::drag-1* and *drag-1p::drag-1::Venus::GPI* plasmids were injected at 150 ng/μl with 30 ng/μl of pBSII KS(–) and 20 ng/μl pDP#MM016B.

### Production of antibodies

The RNA sample extracted from wild-type worms was treated with SuperScript III reverse transcriptase (Invitrogen) using a primer 5′-TCAGCATAACAATGATAAAAGAGC-3′ designed to anneal at the 3′-end of the coding region, and single-strand cDNA was produced. The double-strand cDNA was amplified by PCR using a primer designed to anneal at the SL1 splice leader sequence 5′-GGTTTAATTACCCAAGTTTGAG-3′ and the 3′-end primer^48^. The region coding for DRAG-1 peptide from I131 to E368 was amplified using primers 5′-GTCACATATGATAATGTTCAATGGCTCCGTGC-3′ and 5′-GTCACTCGAGTTCTTTCTGGAACCGAGCATG-3′, digested with *Nde*I and *Xho*I, and ligated into the pET-19b vector using the *Nde*I and *Xho*I sites. The resulting antigenic peptide of DRAG-1 was expressed as a histidine-tagged fusion protein in *Escherichia coli* and was used to immunize rabbits. The generated antibody was affinity purified.

### Immunohistochemistry

Immunohistochemistry was performed as described^49^. The DRAG-1 antibody was used as the primary antibody at 4 μg/ml. Alexa 594–labeled donkey anti-rabbit IgG (Life Technologies) was used as the secondary antibody at a dilution of 1:500.

### Microscopy

Nomarski and fluorescence microscopy was performed using an Axioplan 2 microscope equipped with Axiocam CCD camera (Zeiss). Confocal laser scanning microscopy was conducted with LSM5 (Zeiss) equipped with a C-apochromat 63× (water immersion; NA, 1.2) lens controlled by PASCAL version 3.2 SP2 or ZEN software (Zeiss).

## Supplementary Information

Supplementary Information.

## Data Availability

Strains and plasmids are available upon request. The authors state that all data necessary for confirming the conclusions presented in the article are represented fully within the article.
