# Supplementary material for: Repulsive guidance molecule acts in axon branching in Caenorhabditis elegans
Source: Sci Rep. 2021 Nov 16;11:22370. doi: 10.1038/s41598-021-01853-8 (PMC8595726; doi:10.1038/s41598-021-01853-8)
Supplement: Supplementary file 1 — Supplementary Information. [file 41598_2021_1853_MOESM1_ESM.pdf]

Supplementary Information

Repulsive Guidance Molecule Acts in Axon Branching in *Caenorhabditis elegans*

Kaname Tsutsui[1], Hon-Song Kim[1], Chizu Yoshikata[1], Kenji Kimura[1], Yukihiko Kubota[1], Yukimasa Shibata[1], Chenxi Tian[2], Jun Liu[2] & Kiyoji Nishiwaki[1,*]

[1]Department of Bioscience, Kwansei Gakuin University, 2-1 Gakuen, Sanda 669-1337, Japan

[2]Department of Molecular Biology and Genetics, Cornell University, Ithaca, NY 14853, USA

*To whom correspondence should be addressed:

Kiyoji Nishiwaki

Department of Bioscience, Kwansei Gakuin University

2-1 Gakuen, Sanda 669-1337, Japan

Phone: +81-79-565-7639

FAX: +81-79-565-9077

E-mail: nishiwaki@kwansei.ac.jp

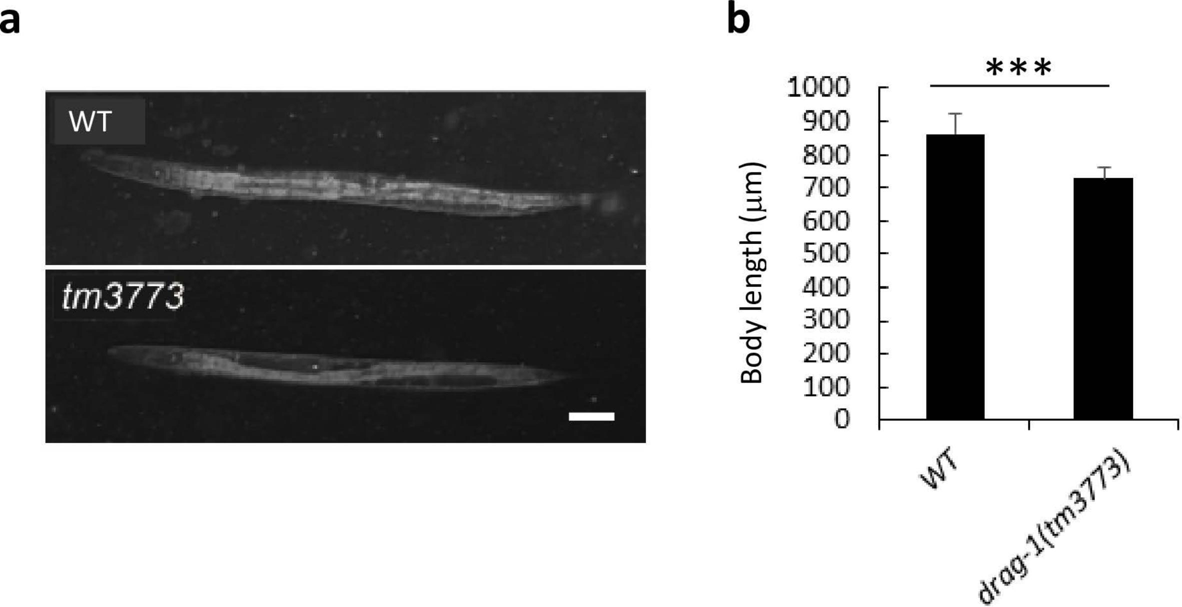

Supplementary Figure S1. Body length phenotype of *drag-1(tm3773)* mutants. (**a**) Body length of young adult hermaphrodites. *tm3773* mutants had shorter bodies compared with wild type. Anterior is to the left. Scale bar: 50 μm. (**b**) Quantification of body length of young adult hermaphrodites for wild-type and *drag-1* mutant animals. Significant difference was determined by Student's t-test. ***$P < 0.001$. $n = 60$ for each strain.

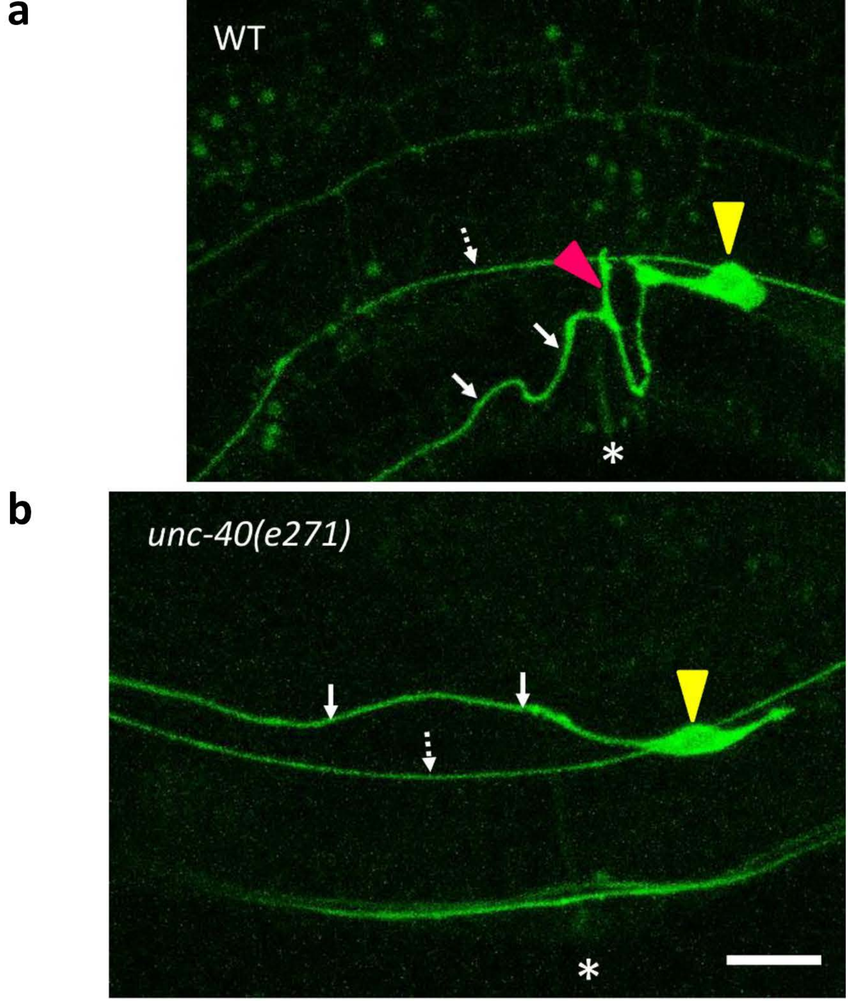

Supplementary Figure S2. Abnormal axon guidance in *unc-40(e271)*. Confocal Z-stack images of wild type (**a**) and *unc-40(e271)* (**b**) young adult hermaphrodites with the *unc-86p::myrGFP* transgene. White arrows and dotted arrows depict the HSN and PLM axons, respectively. Yellow arrowheads and magenta arrowhead depict the HSN cell body and HSN branch, respectively. Anterior to the left, ventral down. The HSN axons in *unc-40(e271)* often extended anteriorly rather than ventrally as those did in the wild type. Asterisk points to vulva. Scale bar: 10 μm.

Supplementary Table S1 Direction of axon protrusion and axon branching in mutants

Genotype	Direction of axon protrusion (%)				w/o branch (%)	number
	ventral	anterior	posterior	multipolar		
wild type	99	0	0	1	24	70
drag-1(tk81)	100	0	0	0	44	62
unc-40(e271)	6	63	27	5	97	64
unc-40(e271)/+	100	0	0	0	35	69
